# Calcium/Calmodulin Kinase IV Controls the Function of Both T Cells and Kidney Resident Cells

**DOI:** 10.3389/fimmu.2018.02113

**Published:** 2018-10-01

**Authors:** Andrew P. Ferretti, Rhea Bhargava, Shani Dahan, Maria G. Tsokos, George C. Tsokos

**Affiliations:** Department of Medicine, Beth Israel Deaconess Medical Center, Harvard Medical School, Boston, MA, United States

**Keywords:** calcium/calmodulin kinase IV, CaMK4, IL-2 deprivation, Treg deficiency, IL-17, podocyte dysfunction

## Abstract

Calcium calmodulin kinase IV (CaMK4) regulates multiple processes that significantly contribute to the lupus-related pathology by controlling the production of IL-2 and IL-17 by T cells, the proliferation of mesangial cells, and the function and structure of podocytes. CaMK4 is also upregulated in podocytes from patients with focal segmental glomerulosclerosis (FSGS). In both immune and non-immune podocytopathies, CaMK4 disrupts the structure and function of podocytes. In lupus-prone mice, targeted delivery of a CaMK4 inhibitor to CD4^+^ T cells suppresses both autoimmunity and the development of nephritis. Targeted delivery though to podocytes averts the deposition of immune complexes without affecting autoimmunity in lupus-prone mice and averts pathology induced by adriamycin in normal mice. Therefore, targeted delivery of a CaMK4 inhibitor to podocytes holds high therapeutic promise for both immune (lupus nephritis) and non-immune (FSGS) podocytopathies.

## Introduction

Calcium/calmodulin-dependent kinase IV (CaMK4) is a serine threonine kinase important for activating transcription factors downstream of T cell receptor (TCR) signaling. Aberrant activation of CaMK4 contributes to T cell abnormalities in systemic lupus erythematosus (SLE), a chronic systemic autoimmune disease presenting with diverse clinical manifestations (1). Immunologic abnormalities are a hallmark in the pathogenesis of SLE including altered antigen receptor–mediated activation and signaling in both T and B cells, defective clearance of immune complexes, neutrophil extracellular traps formation, auto-antibody production, and complement activation (2). Importantly, a multitude of pathways contribute to the expression of SLE pathology and this complicates the identification of a single specific molecule that will result in a successful treatment for all or the majority of the patients. However, many studies suggest that CaMK4 is a central molecule contributing to multiple pathological pathways in T cells from patients with SLE including suppression of IL-2, increased production of IL-17, and imbalance between regulatory and Th17 cells.

## Ca^2+^/calmodulin dependent protein kinases (CaMKs)

Calcium (Ca^2+^) is a ubiquitous universal intracellular second messenger, responsible for the control of numerous cellular processes (3). It exerts its functions by forming a complex with calmodulin (CaM), a 148-amino acid key protein that transduces signals in response to elevation of intracellular Ca^2+^ (4). Ca^2+^ binding to CaM induces conformational changes, leading to increased affinity of the complex for its targets. One such target is CaMK4 (4, 5). The multifunctional CaMK4 has been isolated and localized in the nucleus, with a rather limited normal tissue distribution in discrete regions of the brain, T-lymphocytes, and post-meiotic germ cells (6–8). CaMK4 is activated by the binding of Ca^2+^/CaM causing a structural modification by removing the auto-inhibitory domain exposing the catalytic pocket and enabling substrate access. To be fully activated and gain independent activity, CaMK4 requires phosphorylation on a threonine residue in the activation loop. This is generated by the upstream Ca^2+^/CaM-dependent kinase kinases (CaMKKs) (9).

Upon activation, the autonomous CaMK4 is translocated into the nucleus, where it regulates the activity of several transcription-related components, including cyclic-AMP-response-element-binding protein (CREB), CREB-binding protein, cyclic-AMP response element modulator α (CREMα), histone deacetylase 4, monocyte enhancer factor 2A (MEF2), and retinoid orphan receptor (ROR) (4, 10–16). These factors play a key role in immune system development and function, including regulation of T cell differentiation, cytokines secretion and cell signaling (13, 17–19).

## Contribution of CaMK4 to the suppression of IL-2 production in SLE

Reduced IL-2 is a fundamental immunologic abnormality of T lymphocytes from patients with SLE and mice prone to lupus (20, 21). Since regulatory T (Treg) cells depend highly on IL-2 and IL-2 is diminished in patients with SLE, the number and function of Treg cells is also reduced in SLE patients (22). This skewed cytokine production in SLE also leads to impaired T cell regulation of B cell immunoglobulin production and poor activity of cytotoxic T cells. As a result, SLE patients are predisposed to severe life-threatening infections (23).

**Figure 1:**
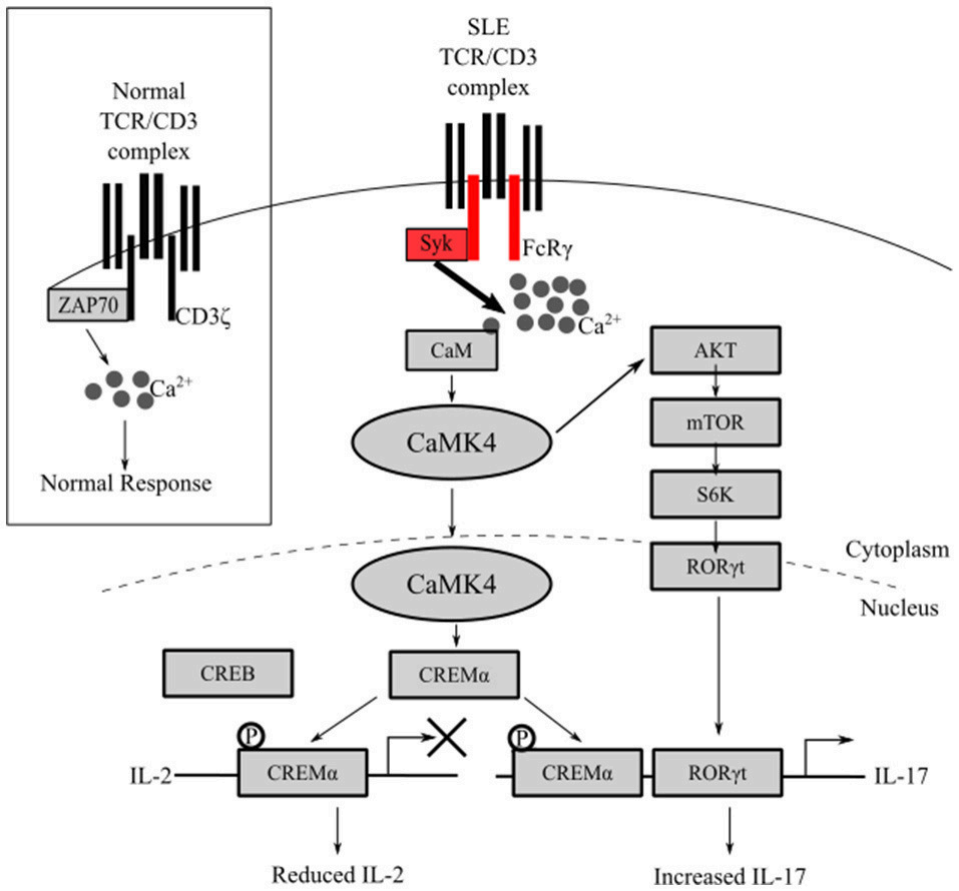
Overactive CaMK4 in T cells signals to reduce IL-2 and increase IL-17 production. In patients with SLE, FcRγ, instead of CD3ζ, associates with the TCR complex and signals through Syk generating a stronger calcium signal. Overactive CaMK4 phosphorylates CREMα and mitigates IL-2 transcription. Concomitantly, CaMK4 promotes IL-17 production through activation of RORγt by phosphorylating AKT.

CaMK4 is a key molecule contributing to reduced IL-2 production in SLE T cells because it controls the ratio of phosphorylated CREB (pCREB) and phosphorylated CREM (pCREM). Activation of the transcription factor CREB by phosphorylation induces IL-2 transcription while activation of CREM by phosphorylation represses IL-2 transcription (24). In T cells from patients with SLE, translocation of CaMKIV to the nucleus is increased. In the nucleus, CaMK4 phosphorylates CREMα, promotes the binding of CREMα to the IL-2 promoter and represses IL-2 transcription (Figure 1) (14). Mechanistically, CREMα recruits DNMT3a and HDAC1 which promote hypermethylation and silencing of gene transcription (25, 26). This mechanism likely contributes to reduced levels of IL-2 in patients with SLE because deletion of CaMK4 reduces pCREMα binding to the IL-2 promoter, restores production of IL-2, and improves *in vitro* polarization of Treg cells. In the MRL/*lpr* lupus-prone mouse, depletion of CaMK4 restores serum levels of IL-2 as well as Treg cell numbers and function (16).

The specific mechanism leading to increased CaMK4 activity in T cells remains nebulous. One potential cause may be the increased calcium signaling in T cells from patients with SLE. Extensive evidence suggests that the CD3 complex in T cells from patients with SLE is rewired to produce an aberrantly enhanced TCR signal (27). Normally, TCR stimulation signals through immune receptor tyrosine-based motifs (ITAM) containing CD3ζ. CD3ζ associates with ZAP70 to propagate the signal. In contrast, T cells from patients with SLE have reduced CD3ζ and the Fc receptor common γ chain (FcRγ) associates with the TCR. Instead of associating with ZAP70, FcRγ associates with spleen tyrosine kinase (Syk) (28). Signaling through FcRγ-Syk in comparison to CD3ζ-ZAP70 transmits a stronger signal leading to enhanced intracellular Ca^2+^ concentration (29, 30). Thus, this enhanced intracellular calcium flux may lead to enhanced CaMK4 activation. Interestingly, in T cells from healthy patients, CaMK4 is activated in response to exposure to IgG isolated from sera from patients with SLE (14). The induction of CaMK4 is attributed to anti-TCR/CD3 antibodies because absorption of serum on TCR/CD3 positive cells but not TCR/CD3 negative cells diminishes CREM binding to the IL-2 promoter. Therefore, in addition to antigen specific activation, activation of the TCR by IgG from patients with SLE potentially contributes to a non-specific activation of T cells.

## Aberrant CaMK4 activation disrupts the balance of treg and Th17 cells in SLE

At the single cell level, the differentiation into either Treg or Th17 cell lineage appears reciprocal in nature (31). For example, upon TCR stimulation, the addition of TGFβ drives naïve T cells to express FoxP3 and differentiate into Treg cells. However, the addition of IL-6 to TGFβ promotes RORγt expression, steers cells toward Th17 differentiation and inhibits FoxP3 and Treg cell differentiation. The reciprocal nature of Treg and Th17 cells is also apparent in SLE where numbers and activity of anti-inflammatory Treg cells are reduced while proinflammatory Th17 cells are increased (32). CaMK4 plays a central role in the imbalance of Treg and Th17 cells. As noted above, CaMK4 contributes to the reduction of IL-2 and limits Treg cells in patients with SLE. Since IL-2 is known to inhibit Th17 differentiation (33), inhibition of IL-2 by CaMK4 likely also promotes Th17 differentiation indirectly.

Furthermore, our lab has established a direct mechanism whereby CaMK4 promotes the polarization of Th17 cells. Increased CaMK4 activity causes increased expression and activity of CREMα in T cells from patients with SLE and lupus-prone mice. Activated CREMα binds to CRE sites in the proximal IL-17 promoter (Figure 1) (34). In contrast to the IL-2 promoter discussed above, CREM binding to the IL-17 promoter facilitates the transcription of IL-17 in T cells from SLE patients (25). An additional pathway whereby CaMK4 promotes Th17 differentiation is through the activation of RORγt (Figure 1). RORγt is a key transcription factor for Th17 differentiation and IL-17 production (13). In T cells from MRL*/lpr* mice, CaMK4 binds to and activates AKT activating the mTOR/S6K pathway (15), a pathway known to activate RORγt. Thus, CaMK4 promotes the differentiation of Th17 cells indirectly by inhibiting IL-2 transcription and directly by promoting IL-17 transcription through CREMα and by activating RORγt through the AKT/mTOR/S6K pathway.

This mechanism is supported by *in vitro* and *in vivo* evidence that suggests CaMK4 promotes Th17 differentiation. In normal T cells, overexpression of CaMK4 increases differentiation of Th17 cells *in vitro* and genetic depletion of CaMK4 disrupts Th17 cell differentiation in T cells derived from normal or autoimmune prone MRL/*lpr* mice (15). Moreover, mice lacking CaMK4 or mice subject to pharmacological inhibition of CaMK4 are resistant to experimental autoimmune encephalomyelitis (EAE), which has been well established that it depends on Th17 cells (15, 35). Thus, ample evidence suggests CaMK4 is a key contributor to IL-17 production and Th17 cell differentiation.

Further evidence suggests CaMK4 activation alters the balance of Treg and Th17 cells in patients with SLE. Activation of CaMK4 is increased in T cells from patients with SLE (14) and lupus-prone MRL/*lpr* mice (16). In T cells from autoimmune-prone MRL/lpr mice, CaMK4 is induced the most after stimulating naïve T cells under Th17 but not Th1, Th2, or Treg polarizing conditions (15). Importantly, depletion of CaMK4 restores IL-2 production (16) and improves Treg cell number and function in MRL*/lpr* mice (36). At the same time, depletion of CaMK4 inhibits Th17 development in SLE T cells (15) and prevents infiltration of IL-17 producing cells in the kidney (35). In sum, increased activation of CaMK4 directs T cells toward Th17 differentiation and away from Treg cell differentiation. Inhibition of CaMKIV restores the Treg/Th17 imbalance, limits lymphocyte proliferation and activation, suppresses nephritis and skin disease, and improves survival in lupus-prone mice (16, 35, 37).

## CaMK4 in resident kidney cells

Lupus nephritis is a major manifestation of SLE occurring in more than 50% of SLE patients and is characterized by immune complex deposition and cell proliferation (38). Resident kidney cells including mesangial cells and podocytes have been implicated in the expression of nephritis in patients with SLE. Interestingly, CaMK4 also plays a role in resident kidney cells and contributes to the pathogenesis of lupus nephritis by promoting mesangial cell proliferation through IL-6 production. Mesangial cells in the glomerulus are known to produce IL-6 when exposed to dsDNA antibodies (39), and in an autocrine fashion, IL-6 stimulates mesangial cell proliferation (40, 41). This is thought to contribute to the pathogenesis of lupus nephritis since blockade of IL-6 or the IL-6 receptor ameliorates kidney disease in lupus-prone mice (42–44). In MRL*/lpr* mice, IL-6 production by mesangial cells in increased, especially upon stimulation with platelet-derived growth factor (PDGF). This increased production is reversed when mice are treated with a CaMK4 inhibitor or genetic depletion of *CaMK4*. Moreover, global depletion of CaMK4 reduces mesangial cell proliferation, and greatly reduces kidney damage (45).

CaMK4 appears to contribute to podocyte disfunction in autoimmune kidney disease. Podocytes from lupus nephritis patients exhibit elevated levels of CaMK4. While the exact mechanism responsible for CaMK4 upregulation is unknown, autoantibodies likely play a role because podocytes exposed to IgG from patients with lupus nephritis display increased CaMK4 and alter the expression of proteins known to be important for the structure and function of podocytes including podocin and nephrin, respectively (46). Also, exposure of podocytes to IgG from patients with SLE causes an increase in the expression of the costimulatory molecules CD80 and CD86 on the surface membrane (46, 47). Global genetic ablation of CaMK4 in MRL*.lpr* mice greatly reduces proteinuria (45).

**Figure 2:**
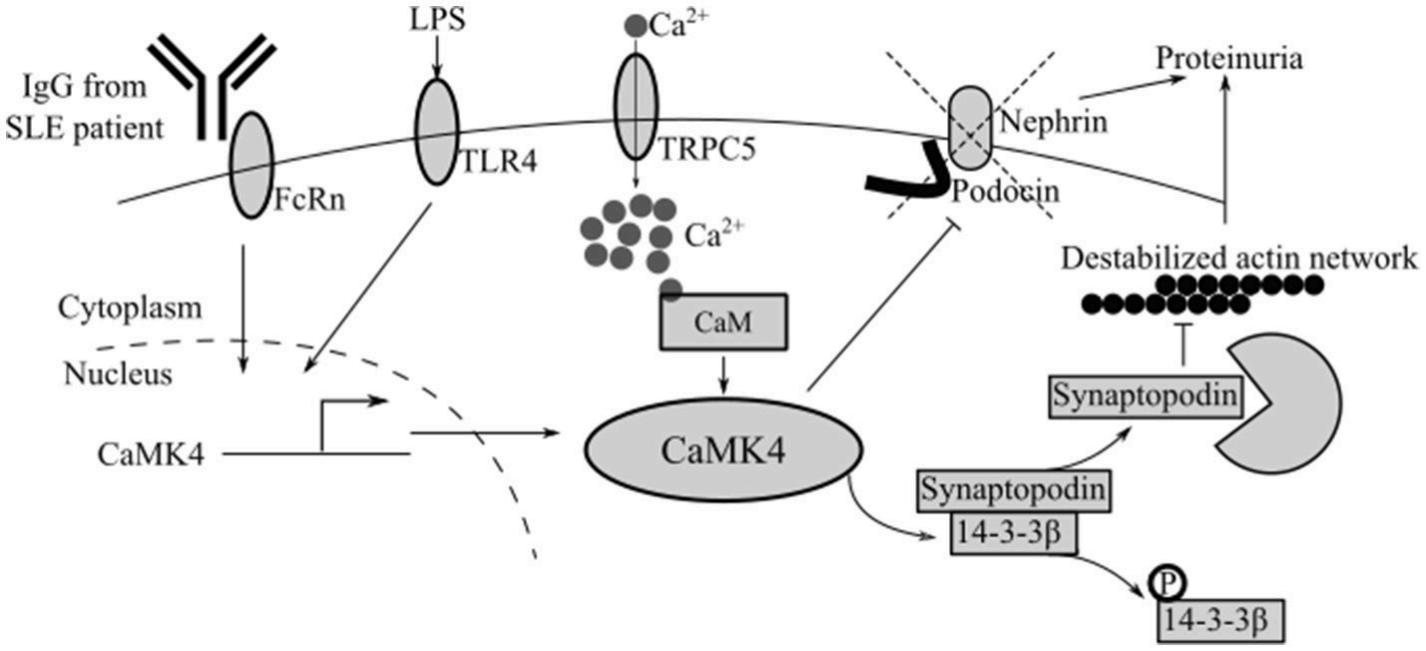
CaMK4 contributes to podocyte dysfunction in autoimmune and non-autoimmune kidney disease. Multiple stimuli including IgG from SLE patients or LPS upregulate CaMK4 leading to destabilization of the actin network and downregulation of nephrin and podocin. TRPC5, short transient receptor potential channel 5.

Podocytes from patients with FSGS also express increased levels of CaMK4 suggesting that this kinase may represent a shared molecule in the expression of immune and non-immune podocytopathies. At the biochemical level, increased levels of CaMK4 disrupt the maintenance of the slit diaphragm by phosphorylating the adaptor molecule 14-3-3β. 14-3-3β stabilizes synaptopodin, an actin binding molecule that is critical for the maintenance of normal actin fiber dynamics. Therefore, phosphorylation of 14-3-3β by CaMK4 causes the release and degradation of synaptopodin leading to destabilization of the actin fiber network (Figure 2) (48).

## Targeting CaMK4 in lupus

To address potential off target concerns of systematic delivery of CaMK4 inhibitors, our lab has explored nanolipogel-based delivery of the CAMK4 inhibitor KN-93 to target CaMK4 specifically in T cells or podocytes. Use of KN-93 packaged nanolipogels coated with an antibody recognizing CD4 targeted KN-93 specifically to CD4 T cells without increasing cell death. Delivery of KN-93 targeted to CD4 T cells to lupus-prone MRL/*lpr* mice increased IL-2 levels in the serum, reduced IL-17 producing infiltrating cells in the kidneys and improved kidney function as measured by proteinuria. Importantly, the effective dose of KN-93 delivered by nanolipogels was 10% of the dose necessary to warrant an effect from systemically delivered KN-93 (35).

Using the same delivery approach, KN-93 targeted to podocytes MRL.*lpr* mice at the beginning of their clinical disease surprisingly never developed proteinuria and immune complexes never deposited despite the fact that humoral and cellular elements of autoimmunity were rampant in these mice. This observation suggests that immune complexes do not deposit if the structure and the function of the podocytes is kept intact. Interestingly, the treated mice did not develop crescents which have been claimed to originate from podocytes (48). Pharmacologic inhibition or silencing of CaMK4 in cultured podocytes subjected to scratch injury did not move to fill up the inflicted empty space.

## Targeting CaMKIV in FSGS

Focal segmental glomerulosclerosis (FSGS) is the most common primary glomerular disease which results in end-stage renal disease. It is a heterogeneous clinical entity characterized by a characteristic histologic pattern. The origin of FSGS is diverse and genetic, metabolic, infectious, and unknown factors have been claimed to be involved in its expression. Proteinuria is the typical clinical finding of FSGS (49). The podocyte is the target cell for injury in FSGS and a growing list of disease-causing gene mutations encoding proteins that regulate podocyte survival and homeostasis has been identified in FSGS patients (50).

Adriamycin has been used extensively to study aspects of FSGS (51). Injection of adriamycin into mice increases the expression of CaMK4 in podocytes. Targeted delivery of a CaMK4 inhibitor to podocytes at the time of injection of adriamycin prevented the development of glomerular damage and more importantly, delivery of the CaMK4 inhibitor 7 days later reversed all damage (48). This evidence strongly urges the consideration of novel approaches to limit FSGS which, through the invariable need of kidney dialysis and transplantation, is responsible for major taxation of the health system expenses.

In summary, CaMK4 is a central molecule that regulates multiple processes that significantly contribute to the pathology of SLE by controlling the production of IL-2 and IL-17 by T cells, the proliferation of mesangial cells and the function and structure of podocytes. CaMK4 is also upregulated in podocytes from patients with FSGS. In both immune and non-immune podocytopathies, CaMK4 disrupts the structure and function of podocytes. It is not known at this point whether CaMK4 is increased in the podocytes from patients with other glomerular diseases and whether it represents a common molecular link for several kidney diseases. It is also not known whether CaMK4 is increased in renal tubular cells in patients with immune and non-immune kidney injury. Although in lupus nephritis it appears that IgG that enters podocytes elicits an increase in the expression of CaMK4, the involved mechanism is still at large. Similarly, we have no insight in to the causes of increased expression of CaMK4 in patients with FSGS although the known increased calcium flux certainly contributes (52). In lupus-prone mice, targeted delivery of a CaMK4 inhibitor suppresses both autoimmunity and the development of nephritis. Yet, targeted delivery to podocytes averts the deposition of immune complexes without affecting autoimmunity. This observation strongly suggests that immune complexes may deposit after podocytes have been injured and changes the approach we should take to prevent kidney damage. It appears that delivery of a CaMK4 inhibitor to podocytes holds high therapeutic promise for both immune (lupus nephritis) and non-immune (FSGS) podocytopathies.

## Author contributions

AF and GT wrote and edited the review. RB, SD, and MT edited the review and aided with review content.

### Conflict of interest statement

The authors declare that the research was conducted in the absence of any commercial or financial relationships that could be construed as a potential conflict of interest.
